# Crystal structures of (*Z*)-(ethene-1,2-di­yl)bis­(di­phenyl­phosphine sulfide) and its complex with Pt^II^ dichloride

**DOI:** 10.1107/S2056989022011847

**Published:** 2023-01-01

**Authors:** Brian Rawls, Jeremy Cunningham, John E. Bender, Richard J. Staples, Shannon M. Biros

**Affiliations:** a Department of Chemistry, Grand Valley State University, Allendale, MI 49401, USA; b Center for Crystallographic Research, Department of Chemistry, Michigan State University, East Lansing, MI 48824, USA; University of Kentucky, USA

**Keywords:** crystal structure, phosphine sulfide, Pt^II^ complex, C—H⋯π inter­action, π–π inter­action, C—H⋯S hydrogen bond, C—H⋯Cl hydrogen bond

## Abstract

The crystal structures of (*Z*)-(ethene-1,2-di­yl)bis­(di­phenyl­phosphine sulfide), C_26_H_22_P_2_S_2_ (**I**), along with its complex with Pt^II^ dichloride, di­chlorido[(*Z*)-(ethene-1,2-di­yl)bis­(di­phenyl­phosphine sulfide)-κ^2^ *S*,*S*′]platinum(II), [PtCl_2_(C_26_H_22_P_2_S_2_)] (**II**), are described here. Compound **I** features P=S bond lengths of 1.9571 (15) and 1.9529 (15) Å, with a torsion angle of 166.24 (7)° between the two phosphine sulfide groups. The crystal of compound **I** features both intra­molecular C—H⋯S hydrogen bonds and π–π inter­actions. Mol­ecules of compound **I** are held together with inter­molecular π–π and C—H⋯π inter­actions to form chains that run parallel to the *z*-axis. The inter­molecular C—H⋯π inter­action has a H⋯*Cg* distance of 2.63 Å, a *D*⋯*Cg* distance of 3.573 (5) Å and a *D*—H⋯*Cg* angle of 171° (where *Cg* refers to the centroid of one of the phenyl rings). These chains are linked by relatively long C—H⋯S hydrogen bonds with *D*⋯*A* distances of 3.367 (4) and 3.394 (4) Å with *D*—H⋯*A* angles of 113 and 115°. Compound **II** features Pt—Cl and Pt—S bond lengths of 2.3226 (19) and 2.2712 (19) Å, with a P=S bond length of 2.012 (3) Å. The Pt^II^ center adopts a square-planar geometry, with Cl—Pt—Cl and S—Pt—S bond angles of 90.34 (10) and 97.19 (10)°, respectively. Mol­ecules of compound **II** are linked in the crystal by inter­molecular C—H⋯Cl and C—H⋯S hydrogen bonds.

## Abstract 2

The crystal structures of the di­sulfide derivative of (*Z*)-ethene-1,2-bis­(di­phenyl­phosphine) as well as its complex with Pt^II^ are described here. The structure of the phosphine sulfide features intra­molecular π–π inter­actions and C—H⋯S hydrogen bonds, as well as inter­molecular π–π and C—H⋯π inter­actions. The structure of the platinum(II) complex features inter­molecular C—H⋯Cl and C—H⋯S hydrogen bonds.

## 1. Chemical context

**Figure 1:**
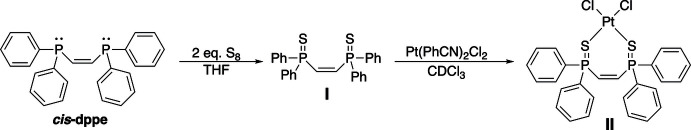
Reaction conditions used to prepare the title compounds.

The diphosphine compound *cis*-bis­(di­phenyl­phos­phino)ethyl­ene (*cis*-dppe, Fig. 1 ▸) has been used by many research groups as a ligand in organometallic chemistry (Hirano & Miura, 2017 ▸; Price & Walton, 1987 ▸). While the bis­phosphine oxide derivative has found use in the coordination chemistry of both *d*-block and *f*-block metals (Jarrett & Sadler, 1991 ▸; Banda & Pritchard, 2008 ▸; Morse, *et al.*, 2016 ▸), the bis­phosphine sulfide and bis­phosphineselenide derivatives have been less studied. Our group is inter­ested in developing new organic compounds that can facilitate the separation of actinide (An) metals from lanthanide (Ln) metals in liquid–liquid extraction processes (Gorden *et al.*, 2013 ▸). Since the An metals have a greater preference for soft-donor atoms than the Ln metals (Cotton, 2006 ▸), there have been some successes with the use of phosphine sulfide compounds as actinide extraction agents (*e.g.* Cyanex 301; Zhu *et al.*, 1996 ▸). To this end, we prepared compound **I** from *cis*-dppe using elemental sulfur (Fig. 1 ▸; Aguiar & Daigle, 1964 ▸; Duncan & Gallagher, 1981 ▸). Unfortunately, our efforts in this area were plagued by the ease of isomerization of the *cis*-alkene to a *trans*-alkene when the systems were heated for even short lengths of time. In an effort to understand the ability of this ligand to form complexes with metals, we also reacted compound **I** with Pt(PhCN)_2_Cl_2_ to give compound **II**. 
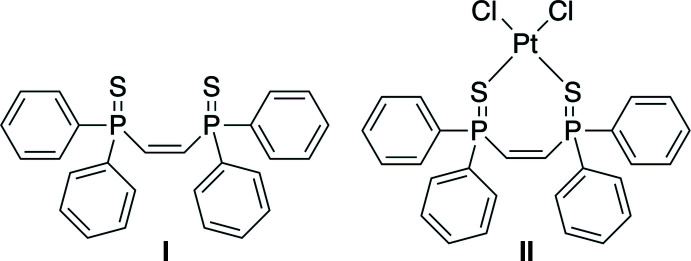


## 2. Structural commentary

**Figure 2:**
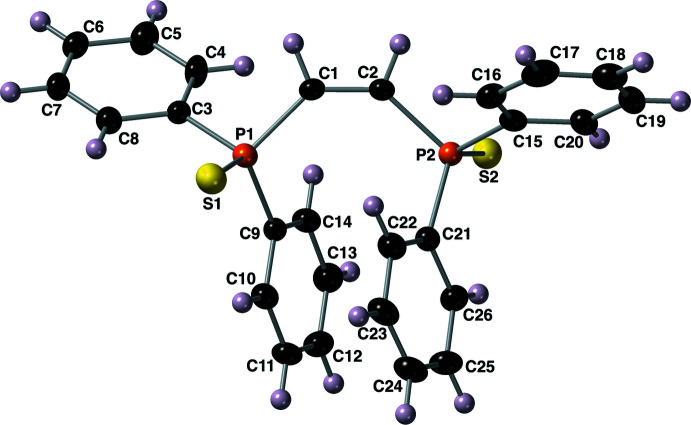
The mol­ecular structure of compound **I**, with the atom-labeling scheme. Displacement ellipsoids are drawn at the 40% probability level using standard CPK colors.

**Figure 3:**
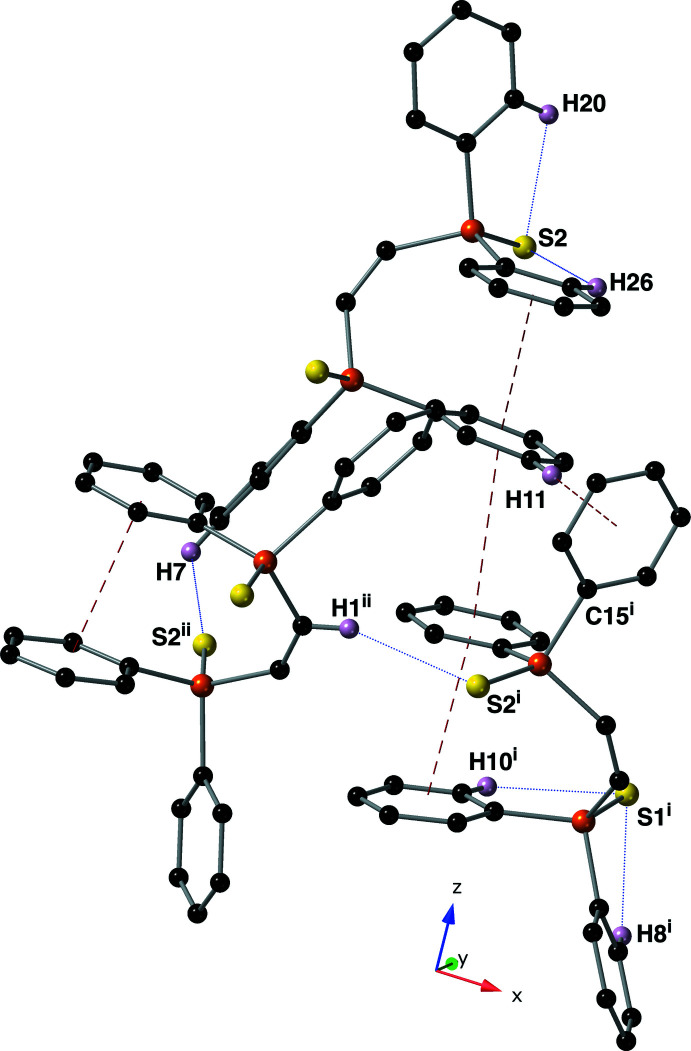
A figure depicting the intra- and inter­molecular inter­actions found in crystals of compound **I** using a ball-and-stick model with standard CPK colors. Hydrogen bonds are drawn using blue dotted lines while π–π and C—H⋯π inter­actions are drawn with red dashed lines. Only hydrogen atoms involved in an inter­action are shown for clarity. Symmetry codes: (i) −*x* + 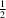 , −*y* + 1, *z* + 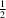 ; (ii) −*x* + 1, *y* − 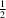 , −*z* + 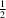 .

The structure of compound **I** was solved in the ortho­rhom­bic space group *P*2_1_2_1_2_1_. The mol­ecular structure of this compound is shown in Fig. 2 ▸ along with the atom numbering scheme. The structure of di­sulfide **I** has P=S bond lengths of 1.9571 (15) and 1.9529 (15) Å, P—C bond lengths that range from 1.804 (4) to 1.824 (4) Å and a C=C bond length of 1.338 (5) Å. The P=S bonds are oriented in opposite directions with a S1—P1—P2—S2 torsion angle of 166.24 (7)°. The τ_4_ descriptor for fourfold coordination around both phospho­rus atoms P1 and P2 is 0.94, indicating a near tetra­hedral geometry of the phosphine sulfide groups (where 0.00 = square-planar, 0.85 = trigonal–pyramidal, and 1.00 = tetra­hedral; Yang *et al.*, 2007 ▸). The bond angles around both phospho­rus atoms range from 100.75 (18) to 115.48 (14)°, with the largest angles involving the sulfur atom. One intra­molecular π–π inter­action is present between the C9–C14 and C21–C26 rings with an inter­centroid distance of 3.737 (3) Å, slippage of 3.370 Å and a dihedral angle of 5.6 (2)°. Both C8(H8) and C10(H10) are engaged in intra­molecular C—H⋯S hydrogen bonds with S1 (Ghosh *et al.*, 2020 ▸; Table 1 ▸). These inter­actions have *D*⋯*A* distances of 3.344 (4) and 3.360 (4) Å with *D*—H⋯*A* dihedral angles of 113 and 116°, respectively (Table 1 ▸, Fig. 3 ▸). In a similar fashion, S2 hosts two intra­molecular C—H hydrogen bonds with C20(H20) and C26(H26). These inter­actions have *D*⋯*A* distances of 3.367 (4) and 3.394 (4) Å with *D*—H⋯*A* dihedral angles of 113 and 115°, respectively. The Flack parameter for this structure is −0.10 (5) (Parsons *et al.*, 2013 ▸).

**Figure 4:**
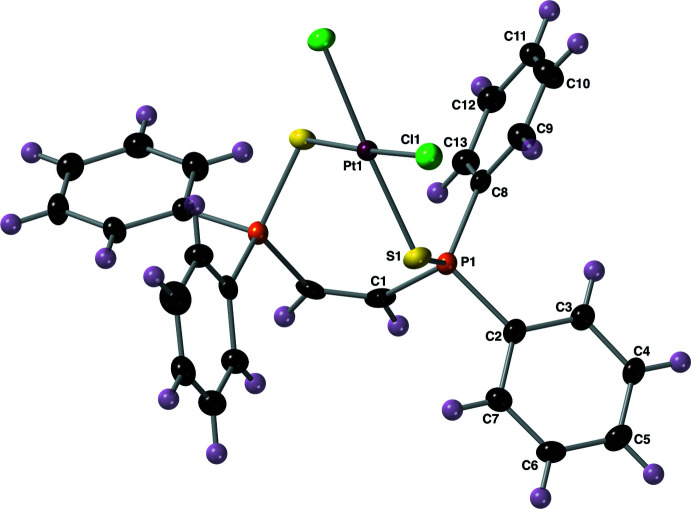
The complete mol­ecular structure of compound **II**, with the atom-labeling scheme. Unlabeled atoms are related to labeled atoms by a crystallographic twofold axis. Displacement ellipsoids are drawn at the 40% probability level using standard CPK colors (Pt = maroon).

For the Pt^II^ complex **II**, the structure was solved in the ortho­rhom­bic space group *Fdd*2. Since the entire mol­ecule straddles a twofold symmetry axis, the asymmetric unit is composed of half of the mol­ecule. The complete mol­ecular structure of compound **II** is shown in Fig. 4 ▸ along with the atom-numbering scheme. The Pt—Cl and Pt—S bond lengths are 2.3226 (19) and 2.2712 (19) Å, respectively. The Cl1—Pt1—Cl1^i^ and S1—Pt1—S1^i^ bond angles are 90.34 (10) and 97.19 (10)°, respectively [symmetry code: (i) −*x* + 1, −*y* + 1, *z*]. The τ_4_ descriptor for fourfold coordination around the Pt^II^ center is 0.05, indicating a nearly perfect square-planar orientation of the sulfur and chlorine atoms around the metal (Yang *et al.*, 2007 ▸). The P=S bond length is 2.012 (3) Å, which is slightly longer than what was observed for compound **I**. The complex has P—C bond lengths that range from 1.799 (8) to 1.816 (9) Å, with a C=C bond length of 1.312 (18) Å. The τ_4_ descriptor for fourfold coordination of the phospho­rus atom P1 is 0.91, indicating a slightly distorted tetra­hedral geometry of the groups bonded to this atom, and that this tetra­hedron is more distorted than what was observed for compound **I**.

## 3. Supra­molecular features

**Figure 5:**
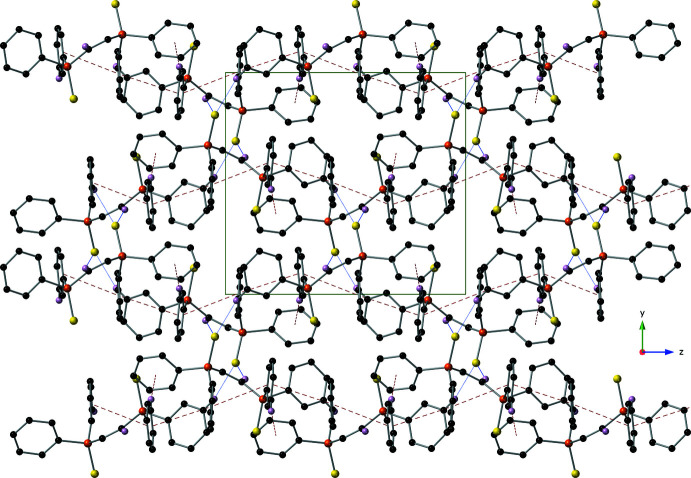
A packing diagram of compound **I** viewed down the *x*-axis using a ball-and-stick model with standard CPK colors. All π–π and C—H⋯π inter­actions are drawn with red dashed lines and inter­molecular C—H⋯S hydrogen bonds are drawn with blue dotted lines. Intra­molecular C—H⋯S hydrogen bonds and any hydrogen atom not involved in an inter­action have been omitted for clarity.

Mol­ecules of compound **I** are held together in the crystal by inter­molecular π–π and C—H⋯π inter­actions (Table 1 ▸ and Fig. 3 ▸). Ring C9–C14 is engaged in an inter­molecular π–π inter­action with a screw-related C21–C26 ring (symmetry code: −*x* + 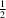 , −*y* + 1, *z* − 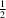 ). The centroid–centroid distance of this inter­action is 3.896 (3) Å, with a slippage of 3.598 Å and a dihedral angle of 10.80 (14) °. Hydrogen atom C11(H11) is engaged in an inter­molecular C—H⋯π inter­action with ring C15–C20 (symmetry code −*x* + 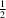 , −*y* + 1, *z* + 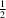 ) with an H⋯*Cg* distance of 2.63 Å, a *D*⋯*Cg* distance of 3.573 (5) Å and a *D*—H⋯*Cg* angle of 171° (*Cg* is the centroid of the C15–C20 ring). Together, these inter­molecular π–π and C—H⋯π inter­actions link the mol­ecules into chains that propagate parallel to the *z*-axis (Fig. 5 ▸). Two potential inter­molecular C—H⋯S inter­actions exist between C1(H1) and C7(H7) and S2. These inter­actions have relatively long *D*⋯*A* distances of 3.742 (4) and 3.561 (5) Å with *D*—H⋯*A* angles of 155 and 135°, respectively. These hydrogen-bonding inter­actions occur between the supra­molecular chains of compound **I**.

**Figure 6:**
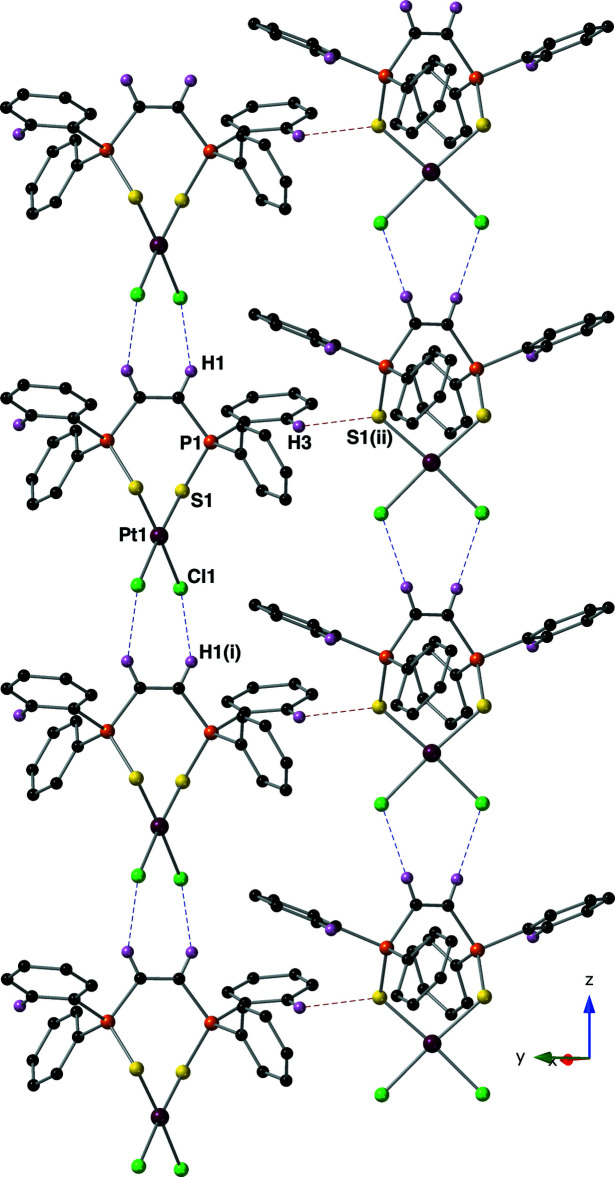
This figure shows the inter­molecular C—H⋯Cl and C—H⋯S hydrogen bonds present in the crystal of compound **II** using a ball-and-stick model with standard CPK colors (Pt = maroon). Hydrogen bonds are drawn with blue (C—H⋯Cl) or red (C—H⋯S) dashed lines, and only hydrogen atoms H1 and H3 are shown for clarity. Symmetry codes: (i) −*x* + 1, −*y* + 1, *z* − 1; (ii) *x* + 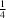 , −*y* + 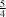 , *z* + 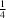 .

Mol­ecules of compound **II** are held together by C—H⋯Cl (Aullón *et al.*, 1998 ▸) and C—H⋯S hydrogen bonds (Ghosh *et al.*, 2020 ▸; Table 2 ▸ and Fig. 6 ▸). The C—H⋯Cl inter­action is between hydrogen atom C1(H1) and Cl1 and has a *D*⋯A distance of 3.515 (10) Å with a *D*—H⋯*A* angle of 141° (symmetry code: −*x* + 1, −*y* + 1, *z* + 1). Sulfur atom S1 hosts the other inter­molecular hydrogen bond with atom C3(H3) (symmetry code: *x* + 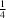 , −*y* + 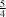 , *z* + 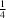 ). This inter­action has a slightly longer *D*⋯A distance of 3.538 (9) with a *D*—H⋯*A* angle of 133°. The inter­molecular C—H⋯Cl inter­actions form chains of compound **II** that run parallel to the *z*-axis. These chains are then linked into a three-dimensional network through the inter­molecular C—H⋯S hydrogen bonds.

## 4. Database survey

A search of the Cambridge Structural Database (CSD version 5.42, Sep. 2021; Groom *et al.*, 2016 ▸) for structures similar to compound **I** resulted in 17 hits. The majority of these hits were metal–ligand complexes, where the ligand was a triazole ring bearing two di­phenyl­phosphine sulfide groups. Crystal structures of this ligand bonded to copper(II), zinc(II), palladium(II), and cadmium(II) were reported (KOBJOC, KOBKAP, KOBKUJ, KOBKIX; Pastor-Medrano, *et al.*, 2014 ▸), along with complexes containing zirconium(IV) and hafnium(IV) (PUKNAM, PUKNEQ; Bernabe-Pablo *et al.*, 2016 ▸). A structure closely related to compound **I**, where the alkene bears a phenyl ring and is bonded to the phosphine sulfide groups with a *trans* relationship, has also been deposited in the CSD as a private communication (GOLXAI; Rybakov and Afanas’ev, 2010 ▸).

## 5. Synthesis and crystallization

Compound **I**: *cis*-dppe (500 mg, 1.25 mmol) and elemental sulfur (S_8_, 80 mg, 0.31 mmol) were combined in a round-bottom flask and dissolved in tetra­hydro­furan (5 mL). The reaction mixture was stirred for three h at room temperature. The solvent was removed under reduced pressure to give a white, gelatinous solid. The crude product was recrystallized from benzene (5 mL) at 333 K and isolated by vacuum filtration with a Hirsch funnel to give a white solid. Analysis of the solid by ^31^P NMR (CDCl_3_) showed that the target compound **I** was present along with *trans*-dppeS_2_ and unreacted starting material. Single crystals of compound **I** grew serendipitously upon slow evaporation of this solution. ^31^P NMR (CDCl_3_, 121 MHz): Compound **I**: 32.3 ppm; *trans*-dppeS2: 36.6 ppm; *cis*-dppe: −22 ppm.

Compound **II**: Equimolar amounts of compound **I** (10.0 mg, 0.022 mmol) and Pt(PhCN)_2_Cl_2_ (10.4 mg, 0.022 mmol) were combined in a small vial and dissolved in 1 mL CDCl_3_. Crystals of compound **II** formed serendipitously *via* slow evaporation of the solvent.

## 6. Refinement

Crystal data, data collection and structure refinement details are summarized in Table 3 ▸. For compounds **I** and **II**, all hydrogen atoms bonded to carbon atoms were placed in calculated positions and refined as riding: C—H = 0.95–1.00 Å with *U*_iso_(H) = 1.2*U*_eq_(C) for vinylic and aromatic hydrogen atoms.

## Supplementary Material

Crystal structure: contains datablock(s) I, II. DOI: 10.1107/S2056989022011847/pk2674sup1.cif

Structure factors: contains datablock(s) I. DOI: 10.1107/S2056989022011847/pk2674Isup4.hkl

Structure factors: contains datablock(s) II. DOI: 10.1107/S2056989022011847/pk2674IIsup5.hkl

CCDC references: 1481532, 2226172

Additional supporting information:  crystallographic information; 3D view; checkCIF report

## Figures and Tables

**Table 1:** Hydrogen-bond geometry (Å, °) for **I** [Chem scheme1] *Cg* is the centroid of the C15–C20 ring.

*D*—H⋯*A*	*D*—H	H⋯*A*	*D*⋯*A*	*D*—H⋯*A*
C1—H1⋯S2^i^	0.95	2.86	3.742 (4)	155
C7—H7⋯S2^ii^	0.95	2.82	3.561 (5)	135
C8—H8⋯S1	0.95	2.86	3.344 (4)	113
C10—H10⋯S1	0.95	2.84	3.360 (4)	116
C20—H20⋯S2	0.95	2.89	3.367 (4)	113
C26—H26⋯S2	0.95	2.89	3.394 (4)	115
C11—H11⋯*Cg* ^iii^	0.95	2.63	3.573 (5)	171

Symmetry codes: (i) 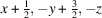 ; (ii) 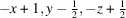 ; (iii) 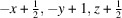 .

**Table 2:** Hydrogen-bond geometry (Å, °) for **II** [Chem scheme1]

*D*—H⋯*A*	*D*—H	H⋯*A*	*D*⋯*A*	*D*—H⋯*A*
C1—H1⋯Cl1^i^	0.95	2.73	3.515 (10)	141
C3—H3⋯S1^ii^	0.95	2.82	3.538 (9)	133

Symmetry codes: (i) 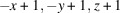 ; (ii) 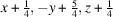 .

**Table 3:** Experimental details

	**I**	**II**
Crystal data		
Chemical formula	C_26_H_22_P_2_S_2_	[PtCl_2_(C_26_H_22_P_2_S_2_)]
*M*_r_	460.49	726.48
Crystal system, space group	Orthorhombic, *P*2_1_2_1_2_1_	Orthorhombic, *F* *d* *d*2
Temperature (K)	173	173
*a*, *b*, *c* (Å)	12.315 (3), 13.092 (3), 14.211 (4)	18.0724 (12), 30.163 (2), 9.3697 (6)
*V* (Å^3^)	2291.2 (10)	5107.5 (6)
*Z*	4	8
Radiation type	Mo *K*α	Mo *K*α
μ (mm^−1^)	0.38	6.01
Crystal size (mm)	0.22 × 0.17 × 0.11	0.35 × 0.14 × 0.10

Data collection		
Diffractometer	Bruker APEXII CCD	Bruker APEXII CCD
Absorption correction	Multi-scan (*SADABS*; Krause *et al.*, 2015 ▸)	Multi-scan (*SADABS*; Krause *et al.*, 2015 ▸)
*T*_min_, *T*_max_	0.655, 0.745	0.543, 0.745
No. of measured, independent and observed [*I* > 2σ(*I*)] reflections	18678, 4213, 3676	20596, 2324, 2227
*R*_int_	0.057	0.039
(sin θ/λ)_max_ (Å^−1^)	0.603	0.603

Refinement		
*R*[*F* ^2^ > 2σ(*F* ^2^)], *wR*(*F* ^2^), *S*	0.038, 0.088, 1.04	0.023, 0.061, 1.09
No. of reflections	4213	2324
No. of parameters	271	150
No. of restraints	0	1
H-atom treatment	H-atom parameters constrained	H-atom parameters constrained
Δρ_max_, Δρ_min_ (e Å^−3^)	0.37, −0.20	1.82, −0.48
Absolute structure	Flack *x* determined using 1430 quotients [(*I* ^+^)−(*I* ^−^)]/[(*I* ^+^)+(*I* ^−^)] (Parsons *et al.*, 2013 ▸)	Flack *x* determined using 1010 quotients [(*I* ^+^)−(*I* ^−^)]/[(*I* ^+^)+(*I* ^−^)] (Parsons *et al.* 2013 ▸)
Absolute structure parameter	−0.10 (5)	−0.006 (4)

Computer programs: *APEX2* and *SAINT* (Bruker, 2013 ▸), *OLEX2.solve* (Bourhis *et al.*, 2015 ▸), *SHELXS* (Sheldrick, 2008 ▸), *SHELXL2019/2* (Sheldrick, 2015 ▸), *OLEX2* (Dolomanov *et al.*, 2009 ▸; Bourhis *et al.*, 2015 ▸), and *CrystalMaker* (Palmer, 2007 ▸).

## supplementary crystallographic information

### (Z)-(Ethene-1,2-diyl)bis(diphenylphosphine sulfide (I) Crystal data

**Table:**

C_26_H_22_P_2_S_2_	*D*_x_ = 1.335 Mg m^−^^3^
*M**_r_* = 460.49	Mo *K*α radiation, λ = 0.71073 Å
Orthorhombic, *P*2_1_2_1_2_1_	Cell parameters from 7213 reflections
*a* = 12.315 (3) Å	θ = 2.2–25.3°
*b* = 13.092 (3) Å	µ = 0.38 mm^−^^1^
*c* = 14.211 (4) Å	*T* = 173 K
*V* = 2291.2 (10) Å^3^	Chunk, yellow
*Z* = 4	0.22 × 0.17 × 0.11 mm
*F*(000) = 960	

### (Z)-(Ethene-1,2-diyl)bis(diphenylphosphine sulfide (I) Data collection

**Table:**

Bruker APEXII CCD diffractometer	3676 reflections with *I* > 2σ(*I*)
φ and ω scans	*R*_int_ = 0.057
Absorption correction: multi-scan (SADABS; Krause *et al.*, 2015)	θ_max_ = 25.4°, θ_min_ = 2.1°
*T*_min_ = 0.655, *T*_max_ = 0.745	*h* = −14→14
18678 measured reflections	*k* = −15→15
4213 independent reflections	*l* = −17→17

### (Z)-(Ethene-1,2-diyl)bis(diphenylphosphine sulfide (I) Refinement

**Table:**

Refinement on *F*^2^	Hydrogen site location: inferred from neighbouring sites
Least-squares matrix: full	H-atom parameters constrained
*R*[*F*^2^ > 2σ(*F*^2^)] = 0.038	*w* = 1/[σ^2^(*F*_o_^2^) + (0.0374*P*)^2^ + 0.5294*P*] where *P* = (*F*_o_^2^ + 2*F*_c_^2^)/3
*wR*(*F*^2^) = 0.088	(Δ/σ)_max_ < 0.001
*S* = 1.04	Δρ_max_ = 0.37 e Å^−^^3^
4213 reflections	Δρ_min_ = −0.20 e Å^−^^3^
271 parameters	Absolute structure: Flack *x* determined using 1430 quotients [(*I*^+^)-(*I*^-^)]/[(*I*^+^)+(*I*^-^)] (Parsons *et al.*, 2013)
0 restraints	Absolute structure parameter: −0.10 (5)
Primary atom site location: iterative	

### (Z)-(Ethene-1,2-diyl)bis(diphenylphosphine sulfide (I) Special details

**Table:**

Geometry. All esds (except the esd in the dihedral angle between two l.s. planes) are estimated using the full covariance matrix. The cell esds are taken into account individually in the estimation of esds in distances, angles and torsion angles; correlations between esds in cell parameters are only used when they are defined by crystal symmetry. An approximate (isotropic) treatment of cell esds is used for estimating esds involving l.s. planes.

### (Z)-(Ethene-1,2-diyl)bis(diphenylphosphine sulfide (I) Fractional atomic coordinates and isotropic or equivalent isotropic displacement parameters (Å^2^)

**Table:**

	*x*	*y*	*z*	*U*_iso_*/*U*_eq_	
S1	0.55697 (10)	0.38129 (8)	0.13055 (8)	0.0420 (3)	
S2	0.39195 (10)	0.80901 (8)	−0.04312 (8)	0.0398 (3)	
P1	0.53899 (8)	0.52703 (8)	0.15692 (7)	0.0300 (3)	
P2	0.44444 (9)	0.67175 (8)	−0.07181 (7)	0.0281 (2)	
C1	0.6036 (3)	0.6052 (3)	0.0686 (3)	0.0306 (9)	
H1	0.677946	0.617631	0.082123	0.037*	
C2	0.5716 (3)	0.6497 (3)	−0.0115 (3)	0.0297 (9)	
H2	0.631332	0.676857	−0.045572	0.036*	
C3	0.6119 (3)	0.5670 (3)	0.2624 (3)	0.0299 (9)	
C4	0.6408 (4)	0.6679 (3)	0.2775 (3)	0.0415 (11)	
H4	0.621187	0.718677	0.232846	0.050*	
C5	0.6981 (4)	0.6949 (4)	0.3576 (3)	0.0476 (12)	
H5	0.717475	0.764283	0.367629	0.057*	
C6	0.7273 (3)	0.6222 (4)	0.4226 (3)	0.0397 (10)	
H6	0.767874	0.640847	0.476863	0.048*	
C7	0.6973 (3)	0.5225 (4)	0.4085 (3)	0.0445 (12)	
H7	0.716344	0.472267	0.453822	0.053*	
C8	0.6397 (3)	0.4940 (3)	0.3292 (3)	0.0382 (10)	
H8	0.619223	0.424702	0.320333	0.046*	
C9	0.3997 (3)	0.5681 (3)	0.1732 (3)	0.0285 (9)	
C10	0.3184 (3)	0.4950 (3)	0.1773 (3)	0.0358 (10)	
H10	0.336454	0.424670	0.171665	0.043*	
C11	0.2115 (3)	0.5236 (4)	0.1896 (3)	0.0398 (11)	
H11	0.155785	0.473502	0.191199	0.048*	
C12	0.1863 (4)	0.6263 (4)	0.1997 (3)	0.0421 (11)	
H12	0.112958	0.646480	0.208632	0.051*	
C13	0.2669 (4)	0.6990 (3)	0.1967 (3)	0.0406 (11)	
H13	0.248927	0.769140	0.203727	0.049*	
C14	0.3735 (3)	0.6705 (3)	0.1836 (3)	0.0345 (10)	
H14	0.428998	0.720940	0.181615	0.041*	
C15	0.4870 (3)	0.6581 (3)	−0.1938 (3)	0.0322 (9)	
C16	0.5569 (4)	0.5795 (3)	−0.2208 (3)	0.0391 (10)	
H16	0.585269	0.533863	−0.174979	0.047*	
C17	0.5849 (4)	0.5681 (4)	−0.3153 (3)	0.0514 (13)	
H17	0.633985	0.515797	−0.333780	0.062*	
C18	0.5419 (4)	0.6322 (4)	−0.3812 (3)	0.0547 (14)	
H18	0.559414	0.622751	−0.445721	0.066*	
C19	0.4738 (4)	0.7098 (4)	−0.3555 (3)	0.0481 (12)	
H19	0.445533	0.754590	−0.402084	0.058*	
C20	0.4456 (4)	0.7235 (3)	−0.2617 (3)	0.0396 (10)	
H20	0.398261	0.777529	−0.244058	0.047*	
C21	0.3463 (3)	0.5701 (3)	−0.0566 (3)	0.0302 (9)	
C22	0.3761 (4)	0.4679 (3)	−0.0612 (3)	0.0359 (10)	
H22	0.450739	0.449838	−0.063759	0.043*	
C23	0.2974 (4)	0.3922 (3)	−0.0619 (3)	0.0422 (11)	
H23	0.318214	0.322403	−0.064286	0.051*	
C24	0.1897 (4)	0.4180 (4)	−0.0592 (3)	0.0486 (13)	
H24	0.135887	0.366066	−0.060870	0.058*	
C25	0.1589 (4)	0.5196 (4)	−0.0539 (3)	0.0493 (13)	
H25	0.084124	0.537013	−0.051412	0.059*	
C26	0.2363 (3)	0.5953 (3)	−0.0524 (3)	0.0384 (10)	
H26	0.214848	0.664858	−0.048423	0.046*	

### (Z)-(Ethene-1,2-diyl)bis(diphenylphosphine sulfide (I) Atomic displacement parameters (Å^2^)

**Table:**

	*U*^11^	*U*^22^	*U*^33^	*U*^12^	*U*^13^	*U*^23^
S1	0.0477 (7)	0.0281 (5)	0.0503 (6)	0.0058 (5)	0.0037 (6)	−0.0026 (5)
S2	0.0449 (7)	0.0288 (6)	0.0457 (6)	0.0060 (5)	−0.0003 (5)	−0.0047 (5)
P1	0.0292 (6)	0.0273 (5)	0.0335 (6)	0.0022 (5)	0.0029 (5)	0.0011 (4)
P2	0.0285 (5)	0.0265 (5)	0.0293 (5)	0.0007 (5)	0.0001 (5)	−0.0009 (4)
C1	0.023 (2)	0.037 (2)	0.032 (2)	−0.0012 (18)	0.0015 (17)	−0.0013 (18)
C2	0.026 (2)	0.030 (2)	0.032 (2)	−0.0026 (17)	0.0026 (17)	−0.0007 (17)
C3	0.025 (2)	0.033 (2)	0.032 (2)	0.0029 (18)	0.0009 (18)	0.0031 (18)
C4	0.051 (3)	0.034 (2)	0.039 (2)	0.005 (2)	−0.012 (2)	0.007 (2)
C5	0.055 (3)	0.039 (3)	0.050 (3)	0.001 (2)	−0.014 (2)	−0.001 (2)
C6	0.033 (2)	0.054 (3)	0.032 (2)	0.003 (2)	−0.0022 (19)	0.001 (2)
C7	0.035 (2)	0.054 (3)	0.045 (3)	0.005 (2)	−0.003 (2)	0.020 (2)
C8	0.036 (2)	0.036 (2)	0.043 (3)	0.004 (2)	0.001 (2)	0.008 (2)
C9	0.027 (2)	0.033 (2)	0.026 (2)	−0.0001 (18)	0.0009 (17)	0.0013 (17)
C10	0.037 (2)	0.032 (2)	0.038 (2)	−0.002 (2)	0.0031 (19)	0.0012 (19)
C11	0.033 (2)	0.044 (3)	0.043 (2)	−0.010 (2)	0.006 (2)	0.001 (2)
C12	0.026 (2)	0.058 (3)	0.042 (3)	0.007 (2)	0.0074 (19)	−0.001 (2)
C13	0.041 (3)	0.035 (3)	0.046 (3)	0.008 (2)	0.004 (2)	−0.003 (2)
C14	0.032 (2)	0.030 (2)	0.041 (2)	−0.0025 (19)	0.0039 (19)	−0.0013 (19)
C15	0.030 (2)	0.032 (2)	0.034 (2)	−0.0089 (18)	0.0019 (17)	−0.0035 (18)
C16	0.032 (2)	0.047 (3)	0.038 (2)	−0.004 (2)	−0.001 (2)	−0.0075 (19)
C17	0.034 (3)	0.074 (4)	0.047 (3)	−0.003 (2)	0.008 (2)	−0.021 (3)
C18	0.044 (3)	0.087 (4)	0.033 (2)	−0.020 (3)	0.007 (2)	−0.010 (3)
C19	0.048 (3)	0.064 (3)	0.032 (2)	−0.017 (3)	−0.003 (2)	0.009 (2)
C20	0.040 (3)	0.041 (3)	0.038 (2)	−0.008 (2)	0.001 (2)	0.0036 (19)
C21	0.032 (2)	0.034 (2)	0.025 (2)	−0.0023 (18)	0.0013 (17)	−0.0023 (18)
C22	0.039 (2)	0.035 (2)	0.033 (2)	0.000 (2)	0.0060 (19)	−0.0055 (19)
C23	0.051 (3)	0.037 (3)	0.038 (3)	−0.012 (2)	0.008 (2)	−0.004 (2)
C24	0.052 (3)	0.056 (3)	0.038 (3)	−0.026 (3)	0.006 (2)	−0.006 (2)
C25	0.032 (2)	0.068 (3)	0.048 (3)	−0.011 (2)	0.005 (2)	0.000 (3)
C26	0.032 (2)	0.046 (3)	0.038 (2)	0.002 (2)	0.0012 (19)	0.000 (2)

### (Z)-(Ethene-1,2-diyl)bis(diphenylphosphine sulfide (I) Geometric parameters (Å, º)

**Table:**

S1—P1	1.9571 (15)	C12—H12	0.9500
S2—P2	1.9529 (15)	C12—C13	1.376 (6)
P1—C1	1.804 (4)	C13—H13	0.9500
P1—C3	1.824 (4)	C13—C14	1.378 (6)
P1—C9	1.812 (4)	C14—H14	0.9500
P2—C2	1.808 (4)	C15—C16	1.396 (6)
P2—C15	1.819 (4)	C15—C20	1.387 (6)
P2—C21	1.810 (4)	C16—H16	0.9500
C1—H1	0.9500	C16—C17	1.395 (6)
C1—C2	1.338 (5)	C17—H17	0.9500
C2—H2	0.9500	C17—C18	1.365 (7)
C3—C4	1.384 (6)	C18—H18	0.9500
C3—C8	1.389 (6)	C18—C19	1.367 (7)
C4—H4	0.9500	C19—H19	0.9500
C4—C5	1.386 (6)	C19—C20	1.390 (6)
C5—H5	0.9500	C20—H20	0.9500
C5—C6	1.375 (6)	C21—C22	1.389 (6)
C6—H6	0.9500	C21—C26	1.395 (6)
C6—C7	1.372 (6)	C22—H22	0.9500
C7—H7	0.9500	C22—C23	1.386 (6)
C7—C8	1.383 (6)	C23—H23	0.9500
C8—H8	0.9500	C23—C24	1.369 (7)
C9—C10	1.387 (6)	C24—H24	0.9500
C9—C14	1.387 (5)	C24—C25	1.384 (7)
C10—H10	0.9500	C25—H25	0.9500
C10—C11	1.380 (6)	C25—C26	1.375 (6)
C11—H11	0.9500	C26—H26	0.9500
C11—C12	1.387 (6)		
			
C1—P1—S1	111.69 (14)	C11—C12—H12	119.8
C1—P1—C3	101.08 (18)	C13—C12—C11	120.4 (4)
C1—P1—C9	109.73 (18)	C13—C12—H12	119.8
C3—P1—S1	112.42 (14)	C12—C13—H13	119.8
C9—P1—S1	114.89 (14)	C12—C13—C14	120.3 (4)
C9—P1—C3	106.02 (18)	C14—C13—H13	119.8
C2—P2—S2	109.54 (13)	C9—C14—H14	120.1
C2—P2—C15	100.75 (18)	C13—C14—C9	119.8 (4)
C2—P2—C21	113.85 (18)	C13—C14—H14	120.1
C15—P2—S2	112.62 (14)	C16—C15—P2	120.8 (3)
C21—P2—S2	115.48 (14)	C20—C15—P2	119.7 (3)
C21—P2—C15	103.53 (18)	C20—C15—C16	119.4 (4)
P1—C1—H1	112.4	C15—C16—H16	120.1
C2—C1—P1	135.2 (3)	C17—C16—C15	119.8 (4)
C2—C1—H1	112.4	C17—C16—H16	120.1
P2—C2—H2	111.6	C16—C17—H17	120.0
C1—C2—P2	136.7 (3)	C18—C17—C16	119.9 (5)
C1—C2—H2	111.6	C18—C17—H17	120.0
C4—C3—P1	121.8 (3)	C17—C18—H18	119.6
C4—C3—C8	119.2 (4)	C17—C18—C19	120.8 (4)
C8—C3—P1	119.0 (3)	C19—C18—H18	119.6
C3—C4—H4	120.0	C18—C19—H19	119.8
C3—C4—C5	120.1 (4)	C18—C19—C20	120.4 (4)
C5—C4—H4	120.0	C20—C19—H19	119.8
C4—C5—H5	119.7	C15—C20—C19	119.7 (4)
C6—C5—C4	120.6 (5)	C15—C20—H20	120.1
C6—C5—H5	119.7	C19—C20—H20	120.1
C5—C6—H6	120.3	C22—C21—P2	121.7 (3)
C7—C6—C5	119.3 (4)	C22—C21—C26	119.1 (4)
C7—C6—H6	120.3	C26—C21—P2	118.7 (3)
C6—C7—H7	119.5	C21—C22—H22	119.9
C6—C7—C8	121.0 (4)	C23—C22—C21	120.3 (4)
C8—C7—H7	119.5	C23—C22—H22	119.9
C3—C8—H8	120.1	C22—C23—H23	120.0
C7—C8—C3	119.8 (4)	C24—C23—C22	120.0 (4)
C7—C8—H8	120.1	C24—C23—H23	120.0
C10—C9—P1	118.9 (3)	C23—C24—H24	119.9
C10—C9—C14	119.7 (4)	C23—C24—C25	120.2 (4)
C14—C9—P1	121.4 (3)	C25—C24—H24	119.9
C9—C10—H10	119.8	C24—C25—H25	119.9
C11—C10—C9	120.4 (4)	C26—C25—C24	120.2 (4)
C11—C10—H10	119.8	C26—C25—H25	119.9
C10—C11—H11	120.3	C21—C26—H26	119.9
C10—C11—C12	119.4 (4)	C25—C26—C21	120.1 (4)
C12—C11—H11	120.3	C25—C26—H26	119.9
			
S1—P1—C1—C2	92.9 (4)	C4—C5—C6—C7	−1.2 (7)
S1—P1—C3—C4	158.3 (3)	C5—C6—C7—C8	1.0 (7)
S1—P1—C3—C8	−21.4 (4)	C6—C7—C8—C3	0.2 (7)
S1—P1—C9—C10	7.6 (4)	C8—C3—C4—C5	1.0 (7)
S1—P1—C9—C14	−173.9 (3)	C9—P1—C1—C2	−35.7 (5)
S2—P2—C2—C1	96.9 (4)	C9—P1—C3—C4	−75.4 (4)
S2—P2—C15—C16	159.7 (3)	C9—P1—C3—C8	104.9 (3)
S2—P2—C15—C20	−23.4 (4)	C9—C10—C11—C12	−1.3 (6)
S2—P2—C21—C22	−170.6 (3)	C10—C9—C14—C13	−0.8 (6)
S2—P2—C21—C26	17.8 (4)	C10—C11—C12—C13	0.5 (7)
P1—C1—C2—P2	9.2 (7)	C11—C12—C13—C14	0.1 (7)
P1—C3—C4—C5	−178.6 (4)	C12—C13—C14—C9	0.1 (6)
P1—C3—C8—C7	178.4 (3)	C14—C9—C10—C11	1.4 (6)
P1—C9—C10—C11	−180.0 (3)	C15—P2—C2—C1	−144.2 (4)
P1—C9—C14—C13	−179.4 (3)	C15—P2—C21—C22	65.9 (4)
P2—C15—C16—C17	177.4 (3)	C15—P2—C21—C26	−105.8 (3)
P2—C15—C20—C19	−176.7 (3)	C15—C16—C17—C18	−1.7 (7)
P2—C21—C22—C23	−171.3 (3)	C16—C15—C20—C19	0.3 (6)
P2—C21—C26—C25	171.0 (3)	C16—C17—C18—C19	2.0 (7)
C1—P1—C3—C4	39.0 (4)	C17—C18—C19—C20	−1.2 (7)
C1—P1—C3—C8	−140.6 (3)	C18—C19—C20—C15	0.0 (7)
C1—P1—C9—C10	134.4 (3)	C20—C15—C16—C17	0.5 (6)
C1—P1—C9—C14	−47.1 (4)	C21—P2—C2—C1	−34.1 (5)
C2—P2—C15—C16	43.0 (4)	C21—P2—C15—C16	−74.9 (4)
C2—P2—C15—C20	−140.0 (3)	C21—P2—C15—C20	102.0 (3)
C2—P2—C21—C22	−42.5 (4)	C21—C22—C23—C24	0.7 (6)
C2—P2—C21—C26	145.8 (3)	C22—C21—C26—C25	−0.8 (6)
C3—P1—C1—C2	−147.3 (4)	C22—C23—C24—C25	−1.2 (7)
C3—P1—C9—C10	−117.2 (3)	C23—C24—C25—C26	0.6 (7)
C3—P1—C9—C14	61.3 (4)	C24—C25—C26—C21	0.4 (7)
C3—C4—C5—C6	0.2 (7)	C26—C21—C22—C23	0.3 (6)
C4—C3—C8—C7	−1.2 (6)		

### (Z)-(Ethene-1,2-diyl)bis(diphenylphosphine sulfide (I) Hydrogen-bond geometry (Å, º)

Cg is the centroid of the C15–C20 ring.

**Table:**

*D*—H···*A*	*D*—H	H···*A*	*D*···*A*	*D*—H···*A*
C1—H1···S2^i^	0.95	2.86	3.742 (4)	155
C7—H7···S2^ii^	0.95	2.82	3.561 (5)	135
C8—H8···S1	0.95	2.86	3.344 (4)	113
C10—H10···S1	0.95	2.84	3.360 (4)	116
C20—H20···S2	0.95	2.89	3.367 (4)	113
C26—H26···S2	0.95	2.89	3.394 (4)	115
C11—H11···*Cg*^iii^	0.95	2.63	3.573 (5)	171

Symmetry codes: (i) *x*+1/2, −*y*+3/2, −*z*; (ii) −*x*+1, *y*−1/2, −*z*+1/2; (iii) −*x*+1/2, −*y*+1, *z*+1/2.

### Dichlorido[(Z)-(ethene-1,2-diyl)bis(diphenylphosphine sulfide)-κ2S,S']platinum(II) (II) Crystal data

**Table:**

[PtCl_2_(C_26_H_22_P_2_S_2_)]	*D*_x_ = 1.890 Mg m^−^^3^
*M**_r_* = 726.48	Mo *K*α radiation, λ = 0.71073 Å
Orthorhombic, *F**d**d*2	Cell parameters from 9927 reflections
*a* = 18.0724 (12) Å	θ = 2.5–25.4°
*b* = 30.163 (2) Å	µ = 6.01 mm^−^^1^
*c* = 9.3697 (6) Å	*T* = 173 K
*V* = 5107.5 (6) Å^3^	Plate, yellow
*Z* = 8	0.35 × 0.14 × 0.10 mm
*F*(000) = 2816	

### Dichlorido[(Z)-(ethene-1,2-diyl)bis(diphenylphosphine sulfide)-κ2S,S']platinum(II) (II) Data collection

**Table:**

Bruker APEXII CCD diffractometer	2324 independent reflections
Radiation source: sealed tube	2227 reflections with *I* > 2σ(*I*)
Graphite monochromator	*R*_int_ = 0.039
Detector resolution: 8 pixels mm^-1^	θ_max_ = 25.4°, θ_min_ = 2.5°
φ and ω scans	*h* = −21→21
Absorption correction: multi-scan (SADABS; Krause *et al.*, 2015)	*k* = −36→36
*T*_min_ = 0.543, *T*_max_ = 0.745	*l* = −11→11
20596 measured reflections	

### Dichlorido[(Z)-(ethene-1,2-diyl)bis(diphenylphosphine sulfide)-κ2S,S']platinum(II) (II) Refinement

**Table:**

Refinement on *F*^2^	Hydrogen site location: inferred from neighbouring sites
Least-squares matrix: full	H-atom parameters constrained
*R*[*F*^2^ > 2σ(*F*^2^)] = 0.023	*w* = 1/[σ^2^(*F*_o_^2^) + (0.0372*P*)^2^ + 4.1023*P*] where *P* = (*F*_o_^2^ + 2*F*_c_^2^)/3
*wR*(*F*^2^) = 0.061	(Δ/σ)_max_ < 0.001
*S* = 1.09	Δρ_max_ = 1.82 e Å^−^^3^
2324 reflections	Δρ_min_ = −0.47 e Å^−^^3^
150 parameters	Absolute structure: Flack *x* determined using 1010 quotients **[(*I*^+^)-(*I*^-^)]/[(*I*^+^)+(*I*^-^)] (Parsons *et al.* 2013)
1 restraint	Absolute structure parameter: −0.006 (4)
Primary atom site location: heavy-atom method	

### Dichlorido[(Z)-(ethene-1,2-diyl)bis(diphenylphosphine sulfide)-κ2S,S']platinum(II) (II) Special details

**Table:**

Geometry. All esds (except the esd in the dihedral angle between two l.s. planes) are estimated using the full covariance matrix. The cell esds are taken into account individually in the estimation of esds in distances, angles and torsion angles; correlations between esds in cell parameters are only used when they are defined by crystal symmetry. An approximate (isotropic) treatment of cell esds is used for estimating esds involving l.s. planes.

### Dichlorido[(Z)-(ethene-1,2-diyl)bis(diphenylphosphine sulfide)-κ2S,S']platinum(II) (II) Fractional atomic coordinates and isotropic or equivalent isotropic displacement parameters (Å^2^)

**Table:**

	*x*	*y*	*z*	*U*_iso_*/*U*_eq_	
Pt1	0.500000	0.500000	−0.21568 (8)	0.02096 (13)	
S1	0.44478 (10)	0.54577 (6)	−0.0554 (2)	0.0278 (4)	
Cl1	0.55528 (10)	0.45658 (6)	−0.3904 (2)	0.0322 (4)	
P1	0.51249 (11)	0.55887 (7)	0.1097 (2)	0.0232 (4)	
C1	0.5064 (5)	0.5214 (3)	0.2610 (10)	0.027 (2)	
H1	0.513340	0.534545	0.352168	0.032*	
C6	0.3769 (4)	0.6526 (3)	0.2775 (10)	0.0340 (16)	
H6	0.326369	0.654598	0.304722	0.041*	
C4	0.4948 (5)	0.6864 (3)	0.2493 (10)	0.037 (3)	
H4	0.525799	0.711707	0.258970	0.045*	
C5	0.4219 (5)	0.6890 (3)	0.2897 (11)	0.0378 (18)	
H5	0.402617	0.715993	0.326227	0.045*	
C3	0.5238 (5)	0.6471 (3)	0.1941 (10)	0.0334 (18)	
H3	0.573993	0.645788	0.164031	0.040*	
C2	0.4790 (4)	0.6101 (3)	0.1836 (8)	0.0272 (16)	
C7	0.4047 (5)	0.6130 (3)	0.2256 (8)	0.0340 (18)	
H7	0.373423	0.587756	0.218478	0.041*	
C8	0.6089 (4)	0.5641 (2)	0.0639 (8)	0.0263 (17)	
C13	0.6637 (4)	0.5485 (3)	0.1545 (9)	0.0332 (18)	
H13	0.650228	0.534949	0.242200	0.040*	
C9	0.6287 (4)	0.5837 (3)	−0.0631 (9)	0.0358 (19)	
H9	0.591769	0.594278	−0.126754	0.043*	
C12	0.7374 (5)	0.5524 (3)	0.1194 (12)	0.037 (2)	
H12	0.774484	0.541843	0.182621	0.044*	
C11	0.7569 (5)	0.5718 (3)	−0.0084 (11)	0.035 (3)	
H11	0.807545	0.574026	−0.034541	0.043*	
C10	0.7034 (5)	0.5880 (3)	−0.0977 (11)	0.047 (2)	
H10	0.717297	0.602202	−0.184109	0.057*	

### Dichlorido[(Z)-(ethene-1,2-diyl)bis(diphenylphosphine sulfide)-κ2S,S']platinum(II) (II) Atomic displacement parameters (Å^2^)

**Table:**

	*U*^11^	*U*^22^	*U*^33^	*U*^12^	*U*^13^	*U*^23^
Pt1	0.01883 (18)	0.02136 (18)	0.02268 (19)	0.0004 (3)	0.000	0.000
S1	0.0218 (10)	0.0335 (11)	0.0283 (10)	0.0078 (8)	−0.0036 (8)	−0.0072 (8)
Cl1	0.0339 (10)	0.0340 (10)	0.0287 (11)	0.0051 (8)	0.0031 (9)	−0.0066 (8)
P1	0.0256 (9)	0.0202 (9)	0.0238 (10)	−0.0008 (7)	0.0003 (8)	−0.0017 (8)
C1	0.020 (4)	0.045 (4)	0.015 (6)	0.004 (4)	−0.004 (3)	−0.012 (4)
C6	0.024 (4)	0.041 (4)	0.036 (4)	0.006 (3)	0.004 (4)	−0.002 (4)
C4	0.037 (5)	0.023 (4)	0.052 (9)	−0.002 (4)	0.004 (5)	−0.007 (4)
C5	0.036 (4)	0.032 (4)	0.046 (5)	0.009 (3)	0.000 (4)	−0.008 (4)
C3	0.024 (4)	0.028 (4)	0.047 (5)	−0.001 (3)	−0.002 (4)	−0.007 (4)
C2	0.030 (4)	0.024 (4)	0.028 (4)	0.001 (3)	−0.003 (3)	−0.002 (3)
C7	0.031 (5)	0.036 (4)	0.035 (4)	−0.004 (4)	0.006 (3)	−0.003 (3)
C8	0.022 (4)	0.020 (3)	0.037 (5)	−0.002 (3)	−0.002 (3)	−0.008 (3)
C13	0.027 (4)	0.036 (4)	0.037 (5)	0.002 (3)	−0.002 (3)	−0.002 (3)
C9	0.026 (4)	0.039 (5)	0.042 (5)	0.002 (3)	−0.004 (4)	0.005 (4)
C12	0.028 (4)	0.037 (5)	0.045 (6)	0.004 (4)	−0.004 (4)	−0.005 (5)
C11	0.023 (5)	0.033 (5)	0.051 (8)	−0.005 (4)	0.006 (4)	−0.007 (4)
C10	0.040 (5)	0.053 (6)	0.049 (6)	−0.011 (4)	0.009 (4)	0.006 (4)

### Dichlorido[(Z)-(ethene-1,2-diyl)bis(diphenylphosphine sulfide)-κ2S,S']platinum(II) (II) Geometric parameters (Å, º)

**Table:**

Pt1—S1	2.2712 (19)	C5—H5	0.9500
Pt1—S1^i^	2.2712 (19)	C3—H3	0.9500
Pt1—Cl1	2.3226 (19)	C3—C2	1.382 (12)
Pt1—Cl1^i^	2.3226 (19)	C2—C7	1.403 (11)
S1—P1	2.012 (3)	C7—H7	0.9500
P1—C1	1.816 (9)	C8—C13	1.387 (11)
P1—C2	1.799 (8)	C8—C9	1.377 (11)
P1—C8	1.801 (7)	C13—H13	0.9500
C1—C1^i^	1.312 (18)	C13—C12	1.377 (12)
C1—H1	0.9500	C9—H9	0.9500
C6—H6	0.9500	C9—C10	1.394 (12)
C6—C5	1.370 (11)	C12—H12	0.9500
C6—C7	1.384 (11)	C12—C11	1.379 (14)
C4—H4	0.9500	C11—H11	0.9500
C4—C5	1.373 (13)	C11—C10	1.367 (14)
C4—C3	1.397 (12)	C10—H10	0.9500
			
S1—Pt1—S1^i^	97.19 (10)	C2—C3—C4	119.5 (8)
S1^i^—Pt1—Cl1	86.24 (6)	C2—C3—H3	120.3
S1^i^—Pt1—Cl1^i^	176.41 (8)	C3—C2—P1	121.6 (6)
S1—Pt1—Cl1	176.41 (8)	C3—C2—C7	119.5 (7)
S1—Pt1—Cl1^i^	86.24 (6)	C7—C2—P1	118.9 (6)
Cl1—Pt1—Cl1^i^	90.34 (10)	C6—C7—C2	119.9 (7)
P1—S1—Pt1	111.14 (10)	C6—C7—H7	120.0
C1—P1—S1	116.2 (3)	C2—C7—H7	120.0
C2—P1—S1	105.1 (3)	C13—C8—P1	121.0 (6)
C2—P1—C1	102.4 (4)	C9—C8—P1	119.7 (6)
C2—P1—C8	110.0 (4)	C9—C8—C13	119.2 (7)
C8—P1—S1	115.0 (3)	C8—C13—H13	119.5
C8—P1—C1	107.4 (4)	C12—C13—C8	121.1 (8)
P1—C1—H1	115.7	C12—C13—H13	119.5
C1^i^—C1—P1	128.6 (3)	C8—C9—H9	120.2
C1^i^—C1—H1	115.7	C8—C9—C10	119.5 (8)
C5—C6—H6	119.8	C10—C9—H9	120.2
C5—C6—C7	120.3 (7)	C13—C12—H12	120.3
C7—C6—H6	119.8	C13—C12—C11	119.4 (9)
C5—C4—H4	119.7	C11—C12—H12	120.3
C5—C4—C3	120.7 (8)	C12—C11—H11	119.9
C3—C4—H4	119.7	C10—C11—C12	120.1 (8)
C6—C5—C4	120.1 (7)	C10—C11—H11	119.9
C6—C5—H5	119.9	C9—C10—H10	119.7
C4—C5—H5	119.9	C11—C10—C9	120.6 (9)
C4—C3—H3	120.3	C11—C10—H10	119.7
			
S1—P1—C1—C1^i^	35.0 (13)	C3—C4—C5—C6	0.6 (15)
S1—P1—C2—C3	−121.8 (7)	C3—C2—C7—C6	−0.2 (12)
S1—P1—C2—C7	55.4 (7)	C2—P1—C1—C1^i^	148.8 (11)
S1—P1—C8—C13	−143.1 (5)	C2—P1—C8—C13	98.5 (7)
S1—P1—C8—C9	36.7 (7)	C2—P1—C8—C9	−81.6 (7)
P1—C2—C7—C6	−177.5 (7)	C7—C6—C5—C4	0.6 (14)
P1—C8—C13—C12	179.9 (7)	C8—P1—C1—C1^i^	−95.3 (12)
P1—C8—C9—C10	179.5 (7)	C8—P1—C2—C3	2.4 (8)
C1—P1—C2—C3	116.4 (7)	C8—P1—C2—C7	179.7 (6)
C1—P1—C2—C7	−66.4 (7)	C8—C13—C12—C11	−0.5 (13)
C1—P1—C8—C13	−12.1 (7)	C8—C9—C10—C11	1.7 (14)
C1—P1—C8—C9	167.7 (6)	C13—C8—C9—C10	−0.6 (12)
C4—C3—C2—P1	178.6 (7)	C13—C12—C11—C10	1.5 (13)
C4—C3—C2—C7	1.4 (13)	C9—C8—C13—C12	0.0 (12)
C5—C6—C7—C2	−0.8 (13)	C12—C11—C10—C9	−2.1 (14)
C5—C4—C3—C2	−1.6 (14)		

Symmetry code: (i) −*x*+1, −*y*+1, *z*.

### Dichlorido[(Z)-(ethene-1,2-diyl)bis(diphenylphosphine sulfide)-κ2S,S']platinum(II) (II) Hydrogen-bond geometry (Å, º)

**Table:**

*D*—H···*A*	*D*—H	H···*A*	*D*···*A*	*D*—H···*A*
C1—H1···Cl1^ii^	0.95	2.73	3.515 (10)	141
C3—H3···S1^iii^	0.95	2.82	3.538 (9)	133

Symmetry codes: (ii) −*x*+1, −*y*+1, *z*+1; (iii) *x*+1/4, −*y*+5/4, *z*+1/4.
